# Fourier Evaluation of Tracings and Acidosis in Labor (FETAL) Framework: An In-Silico Evaluation of a Spectral-Divergence Method

**DOI:** 10.7759/cureus.113290

**Published:** 2026-07-24

**Authors:** Jacques Balayla

**Affiliations:** 1 Obstetrics and Gynaecology, Lady Davis Institute (LDI), Jewish General Hospital, Montreal, CAN

**Keywords:** birth, cardiotocography, computer-assisted, fetal monitoring, signal processing

## Abstract

Cardiotocography (CTG) is central to intrapartum fetal surveillance, yet visual interpretation remains limited by poor reproducibility and low specificity for fetal acidemia. Spectral analysis may retain clinically relevant information that is lost when fetal heart-rate variability is reduced to a single amplitude measure or a broad frequency-band ratio, while also providing an interpretable input for clinical decision support. We therefore developed the Fourier Evaluation of Tracings and Acidosis in Labor (FETAL) framework and used Monte Carlo simulation to determine whether its proposed spectral feature could be identified before clinical data collection. In each replicate, a synthetic reference template was constructed from the median normalized power spectrum of 30 independently generated reference-like fetal heart-rate signals. Twelve replicates were performed across five prespecified scenarios: null, weak, moderate, and strong spectral shifts, as well as a moderate shift under artifact stress. Each scenario included 30 reference signals and 60 independent test signals. Ten-minute recordings sampled at 4 Hz incorporated drift, autocorrelated noise, decelerations, missingness, and maternal heart-rate contamination. Power spectra estimated using Welch’s method were compared with the reference template using Jensen-Shannon divergence, while deviations in standard deviation and low- to high-frequency ratio served as scalar comparators. Median area under the receiver operating characteristic curve (AUROC) for spectral divergence was 0.504 in the null scenario, 0.466 with weak shifts, 0.778 with moderate shifts, 0.917 with strong shifts, and 0.714 with moderate shifts under artifact stress. The scalar comparators remained near chance across all scenarios. These findings indicate that the FETAL spectral-divergence score can detect graded within-band spectral shifts, remains appropriately uninformative when no usable spectral difference is present, and shows the expected decline in performance under artifact stress. The simulation therefore establishes a locked analytic pipeline, with patient-level validation against umbilical-artery blood gases representing the decisive next step toward clinical implementation.

## Introduction

Continuous cardiotocography (CTG) is the most widely used method of intrapartum fetal surveillance, but its clinical performance is constrained by substantial interobserver disagreement, even under standardized three-tier nomenclature, and by low specificity for clinically important acidemia [1-3]. These limitations have direct clinical consequences: poor specificity may contribute to unnecessary cesarean and operative vaginal deliveries and their attendant maternal morbidity, while limited sensitivity may allow some compromised fetuses to escape timely intervention. Computerized interpretation has not consistently improved neonatal outcomes, and artificial-intelligence approaches remain heterogeneous in design, transparency, and validation [4,5]. There is therefore a need to investigate interpretable and reproducible features that might eventually complement conventional visual CTG assessment. Human studies suggest that fetal acidemia may be accompanied by redistribution of heart-rate variability across frequencies rather than by a simple change in overall amplitude [6-8]. The Fourier Evaluation of Tracings and Acidosis in Labor (FETAL) framework asks whether the normalized Fourier power spectrum of a test tracing differs from a reference spectrum and returns a transparent spectral-divergence feature intended for later evaluation within an intrapartum clinical prediction model. The present study does not determine whether this feature improves prediction beyond established CTG variables, computerized CTG features, or clinician interpretation. No conclusions regarding incremental predictive value, clinical diagnostic accuracy, or clinical benefit can be drawn from an entirely synthetic experiment. Before those questions can be evaluated in patient-level data, however, the computational construct must first be shown to behave as intended and, critically, to remain uninformative when no detectable spectral difference is present. In this report, we sought to accomplish two main objectives. The primary objective was to test three prespecified properties of the spectral-divergence score: chance discrimination when no class difference exists, increasing discrimination as the imposed within-band spectral shift strengthens, and reduced discrimination under artifact stress. The secondary objective was to compare the full-spectrum score with overall standard deviation and the low-/high-frequency power ratio. These objectives concern computational identifiability and falsifiability, not clinical validity, incremental predictive performance, or a causal association between spectral divergence and fetal acidemia.

## Technical report

Theoretical construct and synthetic reference

The reference used in this simulation was not intended to represent a physiologically normal patient tracing. In each Monte Carlo replicate, 30 independently generated reference-like fetal heart-rate signals were subjected to the complete preprocessing pipeline, converted to normalized power spectra, and combined by taking the binwise median. This median normalized spectrum defined the synthetic reference template, Q. Each independent test signal produced a normalized spectrum, P, and the FETAL spectral-divergence score was defined as the Jensen-Shannon divergence between P and Q [9]. Jensen-Shannon divergence was selected because it is symmetric, finite after numerical stabilization, and directly compares two normalized distributions defined on the same frequency grid. In contrast, Kullback-Leibler divergence is asymmetric and may become unstable when spectral bins approach zero. The present study did not seek to establish Jensen-Shannon divergence as superior to all alternative measures of spectral similarity; comparisons with metrics such as Wasserstein distance and cosine similarity remain appropriate for future patient-level development. The reference library was rebuilt independently in every replicate so that uncertainty in the synthetic template contributed to observed performance. No test signal contributed to the reference spectrum against which it was evaluated. The method could therefore fail if preprocessing attenuated the imposed spectral displacement, variation in the reference library obscured it, or artifacts generated greater within-group than between-group variation. The simulation was consequently designed as a falsifiable robustness assessment rather than a demonstration guaranteed by construction. This distinction is important because performance against a synthetic reference cannot establish physiological or clinical validity.

Simulation design and rationale for the spectral perturbations

Twelve Monte Carlo replicates were performed for each of five prespecified scenarios: null, weak shift, moderate shift, strong shift, and moderate shift under artifact stress [10]. Each replicate included 30 independently generated signals for construction of the synthetic reference library and two independent test groups consisting of 30 reference-like and 30 altered signals. Signals represented 10-minute fetal heart-rate recordings sampled at 4 Hz and incorporated baseline drift, two oscillatory components, autoregressive noise, decelerations, short intervals of missingness, and occasional maternal heart-rate contamination. The simulated spectral shifts were not derived from a specific effect size reported in a human study and were not intended to reproduce a validated physiological signature of fetal acidemia. Published observations suggesting that fetal compromise may be accompanied by redistribution of fetal heart-rate variability across frequencies provided the conceptual motivation for examining changes in spectral shape [6-8]. The broad low- and high-frequency framing was therefore informed conceptually by prior fetal heart-rate variability research, whereas the exact peak locations and the magnitudes of the weak, moderate, and strong displacements were selected as controlled mathematical perturbations rather than as quantitatively calibrated estimates of human physiology. In the altered signals, spectral peaks were progressively displaced within the prespecified low- and high-frequency regions while approximate total oscillatory power and broad low-/high-frequency allocation were preserved. This design allowed spectral shape to change without necessarily producing a corresponding change in overall signal amplitude or in the aggregate low-/high-frequency power ratio. The purpose was therefore to determine whether a full-spectrum comparison could detect within-band redistribution that might be missed by scalar summaries such as standard deviation or the low-/high-frequency ratio. The null scenario generated both test groups from the same underlying distribution. The weak-, moderate-, and strong-shift scenarios introduced progressively larger within-band peak displacements. The artifact-stress scenario combined the moderate shift with increased noise, missingness, decelerations, and maternal heart-rate contamination. This structure allowed the spectral-divergence score to remain uninformative under the null condition, fail to detect very small perturbations, improve as spectral separation increased, and lose performance when signal quality deteriorated. These simulated perturbations should not be interpreted as established biological changes caused by fetal acidemia. Their biological validity, direction, magnitude, and consistency must be determined in patient-level intrapartum recordings linked to clinical outcomes. The complete parameter settings used to generate the reference-like and altered signals are provided in the supplementary simulation code.

Signal processing

Values outside the physiologically plausible range of 50-210 beats/min were treated as missing. Signals with more than 20% missing data were rejected, while shorter gaps in retained signals were linearly interpolated. A smooth 60-second baseline was estimated and subtracted to reduce low-frequency baseline drift. Power spectral density was estimated using Welch’s method with 256-sample Hann windows and 50% overlap [11]. Spectral power between 0.02 and 0.80 Hz was retained and normalized to unit mass, allowing each spectrum to be interpreted as a distribution of relative power across the analyzed frequency grid. The FETAL spectral-divergence score was then calculated as the Jensen-Shannon divergence between each test spectrum and the independently generated median synthetic reference spectrum. Two scalar comparator features were evaluated. The first was the absolute deviation in time-domain standard deviation from the corresponding reference value. The second was the absolute deviation in the logarithm of the low-/high-frequency power ratio. These comparators were selected to test whether the simulated within-band redistributions could be detected using overall variability or broad frequency-band allocation rather than the full normalized spectrum.

Statistical evaluation

Discrimination between altered and reference-like test signals was summarized using the area under the receiver operating characteristic curve (AUROC) [12]. An AUROC was calculated separately for each replicate, scenario, and candidate feature. Scenario-specific performance was summarized using the median replicate-level AUROC. The reported 95% empirical Monte Carlo intervals were defined as the 2.5th and 97.5th percentiles of the replicate-specific AUROC values. These intervals were obtained directly from the Monte Carlo replicates and were not estimated using bootstrap resampling. They summarize variability across repeated realizations of the stated synthetic experiment and should not be interpreted as confidence intervals for diagnostic performance in a human population.

Reproducibility and ethical considerations

The simulations and analyses were implemented in Python 3.12 (Python Software Foundation, Wilmington, DE) using NumPy, SciPy, pandas, and scikit-learn [13]. The complete simulation code, preprocessing procedures, parameter settings, random-seed information, and replicate-level outputs are available as online supplementary material. The random-number generation procedure was fixed and documented in the supplementary code to permit reproducible generation of the Monte Carlo replicates. No human participants, identifiable information, clinical recordings, or animal data were used. Institutional research-ethics board review was therefore not required.

Results

**Figure 1:**
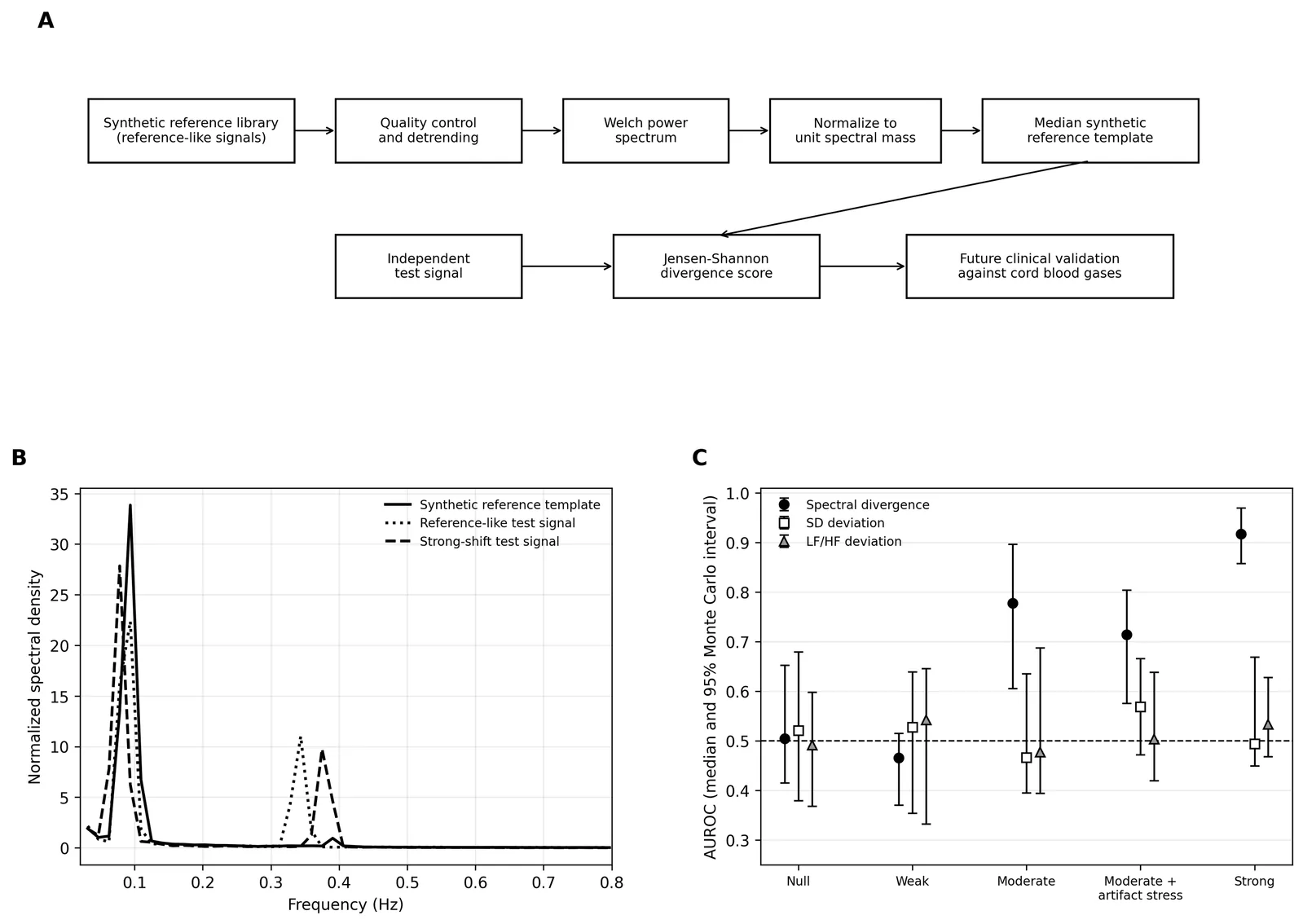
FETAL theoretical framework and falsifiable in-silico evaluation (A) The reference is a median normalized spectrum derived from an independent synthetic reference library. (B) Representative synthetic spectra show the reference template, a reference-like test signal, and a strong-shift test signal. (C) Scenario-specific AUROCs demonstrate chance performance under the null and weak scenarios, improved discrimination with moderate and strong shifts, and degradation under artifact stress. FETAL: Fourier Evaluation of Tracings and Acidosis in Labor; AUROC, area under the receiver operating characteristic curve; LF/HF, low-frequency/high-frequency power ratio; SD, standard deviation.

Across 5,400 generated signals, including 3,600 independent test signals, the median AUROC for spectral divergence was 0.504 (95% empirical Monte Carlo interval, 0.415-0.653) under the null scenario and 0.466 (0.370-0.515) under the weak-shift scenario. Performance increased to 0.778 (0.605-0.896) for moderate shifts and 0.917 (0.858-0.970) for strong shifts. Moderate shifts combined with artifact stress yielded an AUROC of 0.714 (0.575-0.804). Median AUROCs ranged from 0.467 to 0.569 for standard-deviation deviation and from 0.478 to 0.543 for low-/high-frequency-ratio deviation. Figure 1 presents the analytic workflow, representative synthetic spectra, and scenario-specific performance.

## Discussion

The present simulation addresses a deliberately narrow question: whether the FETAL spectral-divergence score behaves coherently and reproducibly under controlled computational conditions. It does not determine whether the score identifies fetal acidemia in clinical recordings, improves prediction beyond established cardiotocographic variables, or adds value beyond clinician interpretation. The findings should therefore be interpreted as evidence of computational identifiability rather than clinical diagnostic validity. Two aspects of the design are central to interpreting the results. First, the reference spectrum was constructed from synthetic reference-like signals generated by the simulation model and was not derived from a cohort of physiologically normal fetuses. Second, the altered spectra were created through controlled redistribution of spectral peaks rather than through a validated biological model of fetal acidemia. Published human studies reporting changes in the distribution of fetal heart-rate variability across frequencies provided the conceptual motivation for examining spectral shape [6-8]. However, the magnitude and precise location of the weak, moderate, and strong shifts were not quantitatively calibrated to effect sizes reported in those studies. The imposed shifts should therefore be understood as mathematical perturbations selected to test the proposed feature under progressively more detectable conditions. Peaks were displaced within prespecified low- and high-frequency regions while approximate total oscillatory power and broad low-/high-frequency allocation were preserved. This design allowed the full normalized spectrum to change without necessarily producing a comparable change in overall standard deviation or the low-/high-frequency power ratio. It consequently tested whether Jensen-Shannon divergence could detect within-band redistribution that scalar summaries might overlook. It does not establish that the simulated patterns reproduce the physiological response to hypoxia or acidemia, and the existence, direction, magnitude, and clinical consistency of such changes must be determined empirically. The method was not guaranteed to discriminate between the simulated groups. Under the null scenario, both test groups arose from the same distribution and the spectral-divergence score remained near chance. The weak-shift scenario was also not reliably distinguishable, indicating that the processing pipeline did not convert every imposed perturbation into apparent discrimination. Performance improved with moderate and strong shifts and declined when the moderate shift was combined with greater artifact burden. These results represent desirable falsification and stress tests: the feature remained uninformative when no usable difference was present, responded progressively when spectral separation increased, and became less reliable when signal quality deteriorated. The strong-shift result must nevertheless be interpreted narrowly. Because the altered signals were generated by relocating spectral peaks and the FETAL spectral-divergence score was designed to quantify full-spectrum divergence, this scenario was favorable to the proposed feature.

The resulting AUROC demonstrates sensitivity to the type of mathematical change the method was designed to detect. It does not demonstrate that acidemic fetuses exhibit this pattern, that the magnitude of the simulated shift is clinically realistic, or that a comparable AUROC would be obtained in humans. The scalar comparators remaining near chance should likewise not be interpreted as evidence that standard deviation or the low-/high-frequency ratio lack clinical value. The simulation was specifically designed to preserve approximate total oscillatory power and broad frequency-band allocation while changing the distribution of power within those bands. The comparators were therefore intentionally less sensitive to the imposed perturbation. Their performance illustrates the distinction between full-spectrum and scalar representations under the stated simulation assumptions, not the comparative clinical superiority of one approach over another.

Jensen-Shannon divergence was used because it provides a symmetric and finite comparison between two normalized spectral distributions on the same frequency grid. These characteristics make the resulting score transparent and straightforward to interpret as a measure of spectral dissimilarity. However, the present study does not establish that Jensen-Shannon divergence is the optimal distributional metric. Future patient-level development should compare it with alternatives such as Kullback-Leibler divergence, Wasserstein distance, cosine similarity, and other measures of spectral shape using prespecified criteria and independent validation. Framed appropriately, this study functions as both an identifiability analysis and a computational unit test. The feature definition, preprocessing pipeline, quality-control rules, spectral estimation procedure, and evaluation scenarios were specified before clinical validation. Null and artifact-stress conditions exposed circumstances in which the method could fail, while the use of independent test signals prevented individual observations from contributing to their own reference template. The documented random-number generation procedure and the availability of the complete simulation code, preprocessing scripts, parameter settings, and replicate-level outputs as supplementary material further support reproducibility. The reported 95% empirical Monte Carlo intervals should not be interpreted as clinical confidence intervals. They represent the 2.5th and 97.5th percentiles of the replicate-specific AUROC values generated under each stated simulation scenario. They therefore summarize variation across repeated realizations of the synthetic experiment rather than sampling uncertainty around diagnostic performance in a human population.

The modest number of replicates also limits the precision with which the tails of these empirical distributions can be characterized. These findings define a hierarchy of evidence for the continued development of the framework. Mathematical coherence asks whether the score is well defined. Computational identifiability asks whether a locked implementation responds to the intended perturbation and remains uninformative under null conditions. Clinical validity asks whether spectral divergence is associated with clinically meaningful outcomes in real intrapartum tracings. Incremental predictive value requires demonstration that the score improves prediction beyond established CTG variables, relevant maternal and fetal characteristics, computerized features, and clinician interpretation. Clinical utility requires further evidence that incorporating the score into decision-making produces a favorable balance of benefits and harms. The present study addresses only the first two levels.

Clinical and public health implications

If subsequently validated in patient-level data, the FETAL spectral-divergence score could potentially serve as an interpretable input to intrapartum clinical decision support. Its intended role would be to complement, rather than replace, visual CTG interpretation. Because it is a transparent, single-valued measure rather than an opaque classifier [14], its contribution to a multivariable model could be examined directly and its relationship with predicted risk could be evaluated for calibration and consistency. However, the present results provide no evidence that adding spectral divergence to established information would improve discrimination, calibration, clinician performance, obstetric intervention rates, or neonatal outcomes. Potential benefits such as reducing unnecessary operative delivery or improving recognition of fetal compromise remain hypothetical. Such claims would require demonstration of reproducible incremental predictive value and clinical utility in appropriately designed human studies. The current simulation establishes only that the feature behaves as intended under specified synthetic conditions, which is a necessary but insufficient step toward clinical use.

Path to clinical validation

Clinical development should use digitized intrapartum CTG recordings linked to paired umbilical-artery blood-gas results [15]. The reference spectrum, preprocessing choices, candidate frequency range, and any model parameters should be developed exclusively within a training cohort. Development and validation must be separated at the patient level so that no windows from the same fetus appear in more than one partition. The spectral-divergence score should first be evaluated as an individual feature and then incorporated into multivariable models containing established CTG characteristics, relevant clinical variables, and, where available, clinician interpretation. A conventional model without spectral divergence should be compared directly with an expanded model containing the feature. This would determine whether the score provides incremental information rather than merely showing an unadjusted association with cord-blood outcomes. Evaluation should include discrimination and calibration [16]. Changes in AUROC alone would be insufficient, particularly if the feature produces only small improvements in ranking. Calibration, clinically relevant sensitivity and specificity, and performance within important subgroups should also be examined. Reporting should follow Transparent Reporting of a Multivariable Prediction Model for Individual Prognosis or Diagnosis Plus Artificial Intelligence (TRIPOD+AI) guidance if the feature is incorporated into a clinical prediction model [17]. Umbilical arterial pH is a defensible initial reference outcome because of its association with adverse neonatal outcomes, although it is not synonymous with neurologic injury and does not fully characterize the clinical condition of the newborn [18]. Decision-curve analysis should then be used to quantify net clinical benefit across plausible decision thresholds [19]. External validation across institutions, patient populations, gestational ages, monitoring devices, and clinical practices would be required before implementation. Future studies may also need to examine complementary outcomes such as base deficit, neonatal encephalopathy, resuscitation requirements, and short-term neonatal morbidity while avoiding data-driven redefinition of endpoints after model evaluation.

Limitations

The generative model was intentionally simplified and does not reproduce the full complexity of real intrapartum cardiotocography. Clinical recordings are affected by nonstationarity, temporal relationships with uterine contractions, fetal sleep and behavioral states [20], gestational-age variation, stage and duration of labor, fetal movement, maternal medications, oxytocin exposure, neuraxial anesthesia, progressive physiological deterioration, and changing baseline and deceleration patterns. Real recordings also contain more complex artifact structures than those represented here. These include prolonged signal loss, abrupt transitions between fetal and maternal heart rates, repeated interpolation, transducer displacement, variable sampling and smoothing algorithms, and differences among external Doppler devices, fetal scalp electrodes, software platforms, and institutions. Such factors could alter normalized spectra, increase within-class variability, or generate divergence unrelated to fetal physiology. The synthetic reference template was not a physiological reference, and the imposed shifts were not empirically estimated signatures of acidemia. The simulation therefore cannot establish which spectral patterns, if any, characterize compromised fetuses. The artifact-stress scenario represented only one prespecified combination of increased noise, missingness, decelerations, and maternal contamination and cannot encompass the full range of signal-quality problems encountered clinically. The number of Monte Carlo replicates was modest, and the empirical intervals describe only variability under the chosen generative assumptions. In addition, although the simulation compared spectral divergence with two scalar summaries, it did not compare the feature with comprehensive computerized CTG models, expert interpretation, or alternative spectral-distance metrics. Most importantly, no human tracings or clinical outcomes were analyzed. The reported AUROCs are therefore not estimates of diagnostic accuracy in patients. No conclusions regarding prediction of fetal acidemia, incremental value beyond established CTG assessment, clinical effectiveness, or improvement in intrapartum outcomes should be drawn from the present study.

## Conclusions

The FETAL spectral-divergence score - the full-spectrum Jensen-Shannon divergence between a tracing’s normalized power spectrum and a synthetic reference spectrum - is an interpretable, single-valued feature whose computational behavior is now characterized. In a prespecified simulation, it remained near chance under the null condition, detected progressively larger imposed within-band spectral shifts, and degraded under artifact stress, while the scalar comparators remained near chance. These findings establish computational identifiability under the stated synthetic assumptions and support locked patient-level validation against paired umbilical-artery blood gases. They do not establish diagnostic performance, incremental predictive value, clinical utility, or improved outcomes in humans.
